# The relationship between sleep and fatigue within community-residing older individuals aged 60 years or above in regional China

**DOI:** 10.3389/fpubh.2026.1880890

**Published:** 2026-07-10

**Authors:** Fei Xu, Qing Ye, Zhiyong Wang, Caihong Hu, Yunting Xu, Guofeng Ao, Luyan Yang, Fengli Li, Shifen Jia, Yu Dou, Guang Li

**Affiliations:** 1 Nanjing Medical University Affiliated Nanjing Municipal Center for Disease Control and Prevention, Nanjing, China; 2 Geriatric Hospital of Nanjing Medical University, Nanjing, China

**Keywords:** aging, fatigability, fatigue, older adults, PFS, sleep

## Abstract

**Objectives:**

To examine associations of sleep quality and duration with physical and mental fatigue among community-residing older inhabitants in Nanjing municipality, China.

**Methods:**

Residents aged 60 years or above were randomly selected from Nanjing in 2023 in this cross-sectional study. The outcome measures, physical and mental fatigue, were evaluated using the validated Chinese version of Pittsburgh Fatigability Scale. The independent measures, sleep quality and duration, were assessed with the Chinese version of Pittsburgh Sleep Quality Index (PSQI) and specific question items, separately. Mixed-effect logistic regression models were used to calculate odds ratios (ORs) and 95% confidence intervals (95%CIs) for investigating associations of sleep quality and duration with fatigue.

**Results:**

Among 5,484 participants, 59.0% (95%CI = 57.7, 63.0) and 51.1% (95%CI = 49.8, 52.4) perceived physical and mental fatigue, separately. After control for confounders, subjects with good PSQI-based sleep quality were less likely to report physical (OR = 0.61, 95%CI = 0.53, 0.70) and mental fatigue (OR = 0.65, 95%CI = 0.56, 0.74) compared to those with poor sleep quality, while no link was observed between self-rated sleep quality and fatigue (OR = 0.88, 95%CI = 0.74, 1.04 for physical fatigue; OR = 0.92, 95%CI = 0.78, 1.09 for mental fatigue). Moreover, the odds were 1.52 (95%CI = 1.26, 1.83), 1.47 (95%CI = 1.26, 1.72), 1.09 (95%CI = 0.94, 1.26) and 1.46 (95%CI = 1.17, 1.81) for experiencing physical fatigue, and 1.60 (95%CI = 1.33, 1.92), 1.43 (95%CI = 1.23, 1.66), 1.07 (95%CI = 0.92, 1.23) and 1.37 (95%CI = 1.11, 1.69) for reporting mental fatigue, respectively, among participants with daily sleep duration of <6.0, 6.0–6.9, 8.0–8.9 and ≥9.0 h relative to those with sleep duration of 7.0–7.9 h/day.

**Conclusion:**

PSQI-based, not self-rated, sleep quality was negatively associated with fatigue, and a U-shape relationship was examined between sleep duration and fatigue among older community-dwelling people in regional China. This study has significant public health implications in this rapidly-aging world for building a healthy-aging society, suggesting that population-level fatigue may be improved through interventions of healthy sleep.

## Introduction

Fatigue, typically described as a self-perceived lack of physical or/and mental energy, is one of the most common complaints in older adults (1–6). Usually, fatigue is an early sign/symptom of unfavorable health conditions for older people (7, 8). It has been investigated that some modifiable factors, including sleep, may be associated with fatigue in older subjects (9, 10). Moreover, sleep problem is also a common health-related issue, with a prevalence of approximately 50%, for older individuals (11, 12). Thus, sleep problem and fatigue are two common issues, and particular attentions shall be paid to sleep-fatigue relationship among older people for building healthy aging societies.

Recently, to improve the comparability of perceived lack of energy in different studies, a new concept, fatigability, was developed to link self-perceived lack of energy to standardized daily activities (4, 13). Obviously, the concept of fatigability is a relatively objective assessment of lack of energy compared to the traditional idea of fatigue, a purely subjective measurement. For simplicity and convenience, these two terms, fatigability and fatigue, are usually used interchangeably to describe the self-perceived lack of energy in academic studies and routine life of ordinary people. To date, the previously-explored sleep-fatigue association was largely among older participants with specific conditions or/and small sample size (14–16), and very few were from community-dwelling older residents (10, 17). For better understanding sleep-fatigue relationship among general older people, it is of necessity to further investigate this issue within representative sample population selected from overall community-residing older adults.

Currently, China has the largest number of older people aged 60 years or above (approximately 264 million in 2020) worldwide (18). However, no studies are available regarding the relationship between sleep and fatigue among large-scale representative community-dwelling older people in China. To address this gap, the present study was developed with aims to investigate the associations of sleep quality and duration, separately, with fatigue among community-residing older residents aged 60 years or above in regional areas of China.

## Methods

### Study design and participants

The cross-sectional data analyzed in this study were derived from the Healthy Aging and Healthy Older Residents study (the HAHOR-fatigue study) that was conducted in Nanjing municipality of China in 2023. There were 12 urban or sub-urban districts and approximately 9.3 million registered inhabitants within Nanjing municipality in 2020 (19). Moreover, among the local residents in Nanjing, 20.9% were those aged 60 years or above (19). The eligible participants were registered residents who were 60 + years old in the whole municipality (20). In detail, a local resident would be eligible to participate in this study, if he/she was: (1) a regular resident registered in Nanjing municipality, (2) 60 + years old, (3) without literal or cognitive/mental problems, (4) able to do daily activities, and (5) not experiencing any active infectious diseases.

Some factors were considered for sample size estimation: (1) the nature of study design (cross-sectional survey), (2) presently available fatigue prevalence (27%) among older people aged 60 years or above (21, 22), (3) the participant’s selection approach (multi-stage sampling), (4) expected statistical power (90%), (5) assumed response rate (90%), and (6) data analysis strategy (stratified by age and gender). Thus, the estimated number of participants would be about 6,000 for the HAHE-fatigue study.

Using a multistage sampling strategy, participants were randomly chosen from the 12 urban and sub-urban districts in Nanjing municipality (23, 24). Considering the five-level governance system in China (From top to bottom: national state, province/municipality, urban district/rural county, street/township and administrative community), two streets/townships were firstly selected from each of the 12 districts in Nanjing Municipality. Then, one administrative community was randomly determined from each chosen street/township, which resulted in totally 24 communities included in the study. Next, according to the estimated numbers of overall participants (N = 6,000) and communities involved (N = 24), 250 eligible participants were expected to be chosen from each determined community. Finally, based on the household list of each community and proportions of overall older residents by age-group (60–69, 70–79, 80 + years = 5.0, 2.3, 1.0) in Nanjing (19), 250 subjects were selected from each involved community.

Written informed consents were obtained from all participants before the study. The ethics approval was granted by the Ethics Committee of Nanjing Medical University Affiliated Nanjing Municipal Center for Disease Control and Prevention. This study was conducted in accordance with guidelines/principles documented in the Declaration of Helsinki.

### Data collection

China National Center for Non-communicable Disease Prevention and Control issued the *Scheme of the Chinese Chronic Non-Communicable Disease (NCD) and Risk Factor Surveillance* to guide data collection for NCD-related population studies in China, in which assessment instruments, experimental procedures, and definitions of variables were recommended (25). The principles and approaches suggested in this Scheme were followed for gathering questionnaire-based information, measuring body weight and height, collecting blood samples, and defining measures in the present study (25).

Via face-to-face interview, information on socio-demographic attributes, behavior/lifestyle, histories of selected NCDs, depressive symptoms, frailty, fatigue and sleep was gathered for each participant with a questionnaire or specific validated sub-scales. Body weight was measured, twice, to the nearest 0.1 kilogram, while body height was assessed to the nearest 0.01 meter two times. Then, the mean values of weight and height were used to compute body mass index (BMI) for each subject (25). For lipid profile assessment, a 5-milliliter fasting venous blood sample was collected from each participant and tested by the designated laboratory using HITACHI7180 analyzer (Hitachi Co., Japan) with detection reagents from Shanghai Fosun Long March Medical Science Co., China (25).

### Study variables

#### Outcome variable

Fatigue was the outcome measure, which was assessed with the validated Chinese version of Pittsburgh Fatigability Scale (PFS-CHN) (26). Specifically, fatigue was evaluated from each of physical and mental dimensions for older people using PFS with consideration of specific standardized daily activities (27). In PFS, physical fatigue was measured with a 10-item sub-scale, while mental fatigue was also assessed using an independent 10-item sub-questionnaire (27). The PFS-CHN performed good for measuring both physical and mental fatigue in terms of validity and reliability among community-residing older people in China (26).

Participants perceived the tiredness, weariness, or/and exhaustion after completion of specific standardized daily activities, and then rated the level of fatigue with a 6-option Likert scale from 0 (no fatigue) to 5 (extreme fatigue) (26, 27). Moreover, the value of each option was correspondingly assigned as 0, 1, 2, 3, 4 or 5. Therefore, the total score ranged from 0 to 50 for physical and mental fatigability, separately. The higher PFS score indicated the greater fatigue perceived by a participant (13, 27). In addition, cutoffs of 15 and 13 were used to classify participants as “lower physical fatigability (physically un-fatigued) (PFS < 15)” or “greater physical fatigability (physically fatigued) (PFS ≥ 15),” and “mentally un-fatigued (PFS < 13)” or “mentally fatigued (PFS ≥ 13),” respectively (13, 27).

#### Independent variable

The independent measures were sleep quality and duration. Sleep quality in the past month was assessed firstly with the validated Chinese version of Pittsburgh Sleep Quality Index (PSQI-CHN) (28, 29). The Chinese version of PSQI was faithfully translated from and shared the scoring approaches with the original PSQI developed and validated by Buysse et al. (30). Seven domains were included in PSQI and each of them was assigned a score range of 0–3, resulting in a total PSQI score of 0–21 for each participant (28–30). However, different from that “>5” was recommended as the cutoff of PSQI score to identify participants with sleep problem (poor sleep quality) in the original validation study by Buysse et al. (30), the optimal cutoff was examined as “>7” to identify subjects with poor sleep quality in Chinese older adults (28, 29). Thus, a population-sensitive PSQI score of “7” was used as the cutoff to classify participants into “poor sleep quality (PSQI>7), the poor sleepers” or “good sleep quality (PSQI≤7), the good sleepers” for analysis in the present study.

Then, sleep quality was self-rated by participants using a simple question of “How did you feel about your sleep quality under the usual circumstance in the past month?,” with four answer options of “excellent, good, poor, or very poor.” For analysis, participants who reported the “poor” or “very poor” sleep quality were grouped as “the poor sleepers,” while those who self-rated “excellent” or “good” sleep quality were categorized as “the good sleepers.” Thus, the associations of both PSQI-based and self-rated sleep quality (classified as “poor” or “good”) with fatigue were examined among participants in this study.

Moreover, typical duration of sleep per day in last month was self-reported by participants with the following question “For a typical day in last month, how many hours of actual sleep did you get (not include the awake time you spend in bed)?.” Then, sleep duration of participants was categorized into: “<6.0,” “6.0–6.9,” “7.0–7.9,” “8.0–8.9,” or “≥9.0 h/day” (31, 32). In this study, “7.0–7.9 h/day” of sleep was used as the reference to investigate the relationship between sleep duration and fatigue among older Chinese adults (31, 32).

#### Covariates

Socio-demographic attributes of participants and potential influencing factors of fatigue were adjusted for in the analysis. The socio-demographic attributes referred to age (60–69, 70–79 or 80 + years old), gender (men or women), residential location (urban or suburban), educational level (≤6, 7–12 or 13 + years of schooling), marriage status (without or with partner/spouse), and occupation category before retirement (domestic worker, service/sales worker, industrial worker, office worker, academic/research staff, or self-employed) (25, 33).

Behavior and lifestyle included drinking, smoking, physical activity (PA), meat and vegetable intake, and body weight status. Participants were classified into “drinkers” or “non-drinkers” and “smokers” or “non-smokers,” separately, based on definitions recommended by China National Center for Non-communicable Disease Prevention and Control (25). PA was measured with the Chinese version of International Physical Activity Questionnaire (IPAQ-CHN) (34, 35). Self-reported seven-day moderate and vigorous PA time was recorded for all participants, respectively. Then, the sum of weekly moderate PA and doubled vigorous PA time was used to categorize participants into “insufficient PA (<150 min/week)” or “sufficient PA (≥150 min/week)” in the analysis (36). Weekly frequencies of meat (red or white meat) and vegetable consumption were assessed with a validated Chinese version of Food Frequency Questionnaire (FFQ) (37). Participants were classified as “recommendation reached” or “recommendation not reached” for meat and vegetable consumption, respectively, based on criteria suggested for older Chinese adults by China Nutrition Society in 2016 (38). Additionally, subject’s body weight status was classified into three categories (“<24 kg/m^2^,” “24-27 kg/m^2^,” or “≥28 kg/m^2^”) according to BMI cutoffs recommended specifically for Chinese adults (39).

Selected chronic conditions were also considered in the multivariate analysis. Information on histories of diabetes, hypertension, chronic obstructive pulmonary disease (COPD), coronary heart disease (CHD), stroke, chronic kidney disease (CKD), and cancer was self-reported as “yes” or “no” by participants (25). Frailty was assessed using the validated Chinese version of Groningen Frailty Indicator (GFI) and participants were classified as “frail (GFI score ≥3)” or “not frail (GFI score <3)” (40, 41), while depressive symptoms were identified using the validated Chinese version of Patient Health Questionnaire-9 (PHQ-9) and subjects were categorized as “negative (PHQ score <5)” or “positive (PHQ score ≥5)” (42, 43). According to objectively-measured level of fasting blood cholesterol, triglyceride, high- and low-density lipoprotein, participant’s lipid profile was treated as “normal” only if all of these four indicators were at normal level, otherwise as “abnormal” (25).

### Statistical analysis

**Figure 1:**
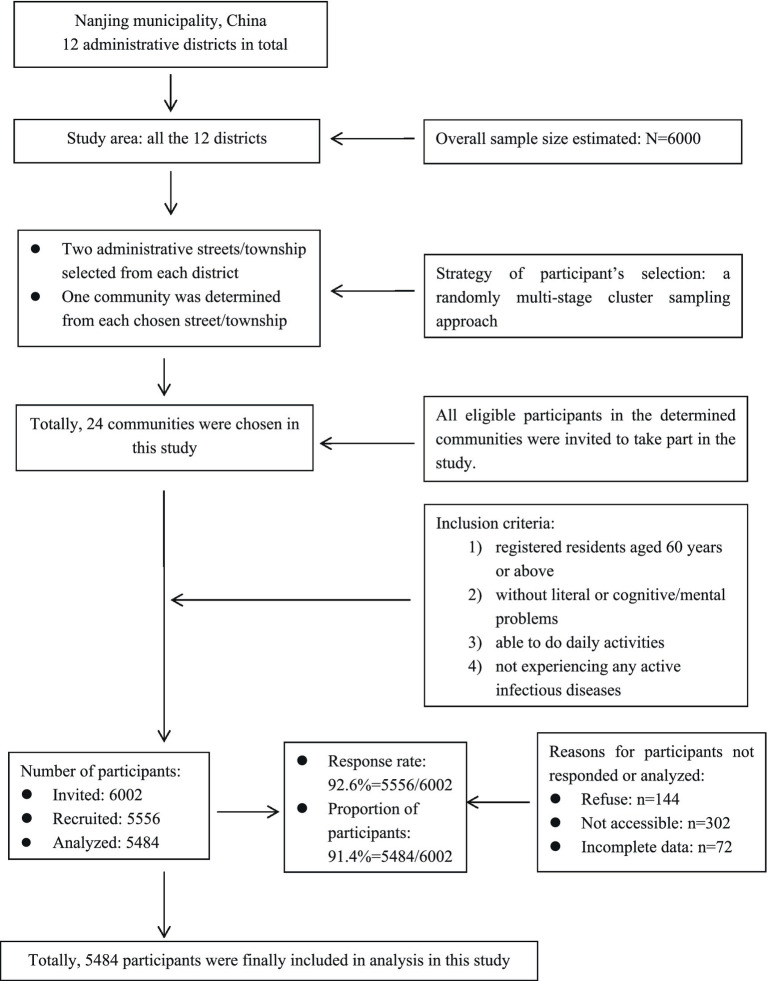
Selection flow chart of participants in this study.

Data were firstly descriptively analyzed to examine differences in the distribution of fatigue (%) between participants’ socio-demographic characteristics using Chi-square test. Next, with mixed-effects logistic regression analysis, odds ratio (OR) together with 95% confidence interval (95%CI) were estimated to examine the associations of sleep quality and duration with fatigue for overall, age−/gender- and area-stratified participants, separately. Model 1 was a univariate logistic regression analysis with sleep quality or duration as the independent measurement and administrative community (the sampling unit) as the random effect. Model 2 was a multivariate logistic regression analysis with further consideration of age (where applicable), gender (where applicable), area (where applicable), education, marriage, occupation before retirement, body weight status, smoking, drinking, PA, meat and vegetable consumption, selected NCDs (diabetes, hypertension, COPD, CHD, stroke, CKD, cancer), frailty, depressive symptoms, and status of lipid profile in addition to those controlled for in Model 1. *p* < 0.05 was treated as the two-sided significance level. SPSS version 20.0 for Windows (SPSS Inc., Chicago, IL, USA) was used to analyze data in the study (Figure 1).

## Results

Totally, 6,002 participants were randomly determined in all the 12 districts of Nanjing municipality, and 5,556 were successfully recruited (sample selection rate = 92.6%). Finally, 5,484 subjects who provided complete information were analyzed in the study. The main reasons for eligible participants not taking part in the survey were “refusal” or “unavailable.” There were no statistical differences in age, gender or residential location between participants included and not included in the analysis. Table 1 displays the selected participants’ attributes by residential location. The 5,484 analyzed participants had a mean age (standard deviation, SD) of 69.7 (6.8), and there were 57.6, 31.2 and 11.2% of them aged 60–69 (the younger-old), 70–79 (the middle-old) and 80 + years (the oldest-old), respectively. Moreover, 49.0% of participants were male older adults and 39.1% resided in urban districts.

**Table 1:** Selected characteristics of participants among men and women in this study (*N* = 5,484).

Characteristics		All	% (n) of participants		*χ* ^2^	*p* value^b^
			Urban^a^	Sub-urban^a^		
Overall		5,484	39.1 (2144)	60.9 (3340)		
Age (years)	60–69	57.6 (3157)	58.5 (1255)	56.9 (1902)		
	70–79	31.2 (1711)	30.3 (650)	31.8 (1061)	1.47	0.478
	80+	11.2 (616)	11.1 (239)	11.3 (377)		
Gender	Men	49.0 (2687)	49.4 (1060)	48.7 (1627)	0.28	0.599
	Women	51.0 (2797)	50.6 (1084)	51.3 (1713)		
Educational attainment (schooling years)	0–6	49.6 (2721)	24.2 (519)	65.9 (2202)		
	7-–12	44.9 (2465)	64.7 (1387)	32.3 (1078)	971.40	<0.001
	13+	5.5 (299)	11.1 (238)	1.8 (60)		
Occupation ^c^	Domestic worker	19.6 (1075)	8.8 (188)	26.6 (887)		
	Industrial worker	42.4 (2327)	33.9 (726)	47.9 (1601)		
	Service/sales worker	6.2 (338)	11.0 (235)	3.1 (103)	906.62	<0.001
	Office worker	8.1 (443)	16.2 (347)	2.9 (96)		
	Academic/research staff	7.0 (384)	13.6 (291)	2.8 (93)		
	Self-employed	16.7 (917)	16.7 (357)	16.7 (560)		
Marital status	Single	13.8 (758)	11.6 (248)	15.3 (510)	15.03	<0.001
	Married/having a partner	86.2 (4726)	88.4 (1896)	84.7 (2830)		
Smoking ^d^	No	76.0 (4168)	78.0 (1673)	74.7 (2495)	7.94	0.005
	Yes	24.0 (1316)	22.0 (471)	25.3 (845)		
Drinking ^e^	No	87.0 (4773)	89.0 (1909)	85.7 (2864)	12.53	<0.001
	Yes	13.0 (711)	11.0 (235)	14.3 (476)		
Body weight status ^f^	BMI < 24	43.5 (2346)	43.3 (930)	42.4 (1416)		
	24 ≤ BMI < 28	42.1 (1130)	41.4 (887)	42.1 (1406)	0.51	0.773
	BMI ≥ 28	14.4 (388)	15.3 (327)	15.5 (518)		

a: Urban and suburban districts were defined based on the official classifications released by China National Bureau of Statistics. b: Chi-square test. c: Occupation referred to job before retirement, and was classified for each participant based on recommendations by China National Center for Chronic Non-communicable Disease Prevention and Control. d: Smoking status was defined as smokers (current- and ex-smokers) and non-smokers (who never smoked cigarettes). e: Drinkers were defined as persons who drank alcohol, on average, at least two times a week for more than 1 year, while non-drinkers were those people who did not meet drinker’s definition. f: Body weight status was categorized based on cutoffs of body mass index (BMI) recommendations for Chinese adults.

Table 2 shows the prevalence of sleep quality and duration among participants by selected characteristics. The proportions of PSQI-based and self-rated good sleeper were 77.7% (95%CI = 76.6, 78.8) and 86.7% (95%CI = 85.8, 87.6), separately, among overall studied older adults in Nanjing municipality of China. The crude agreement rate was 85.0% (4,663/5484) between PSQI-assessed and self-rated sleep quality among participants, while the Kappa value was 0.50 with a moderate consistency between these two predictors in the study. Moreover, 13.4% (95%CI = 12.5, 14.3), 22.9% (95%CI = 21.8, 24.0), 30.3% (95%CI = 29.1, 31.5), 24.8% (95%CI = 23.7, 26.0), and 8.6% (95%CI = 7.9, 9.4) of participants self-reported, respectively, a daily sleep duration of <6.0, 6.0–6.9, 7.0–7.9, 8.0–8.9, and ≥9.0 h. Additionally, when looking at the distribution of sleep attributes by the six main socio-demographic characteristics (age, gender, area, education, occupation, and marriage), difference in PSQI-based sleep quality was not observed only between participants by residential location, while the self-rated sleep quality did not differ for subjects by age, area, education and occupation. As for sleep duration, only gender-disparity was not examined among participants.

**Table 2:** The prevalence of good sleep quality and sleep durations among men and women aged 60 + years by selected characteristics in this study (*N* = 5,484).

Characteristics		% (n/N) of participants with good sleep quality						% (n/N) of participants within categories of sleep time (hour/day)						
		PSQI-based good sleep quality (PSQI≤7)^a^	*χ* ^2^	*p-*value^b^	Self-rated good sleep quality^c^	*χ* ^2^	*p-*value^b^	7.0–7.9	6.0–6.9	8.0–8.9	<6.0	≥9.0	*χ* ^2^	*p-*value^b^
Overall		77.7 (4,263/5484)			86.7 (4,754/5484)			30.3 (1,664/5484)	22.9 (1,255/5484)	24.8 (1,359/5484)	13.4 (735/5484)	8.6 (471/5484)		
Age (years)	60–69	80.1 (2,528/3157)			87.0 (2,748/3157)			31.5 (996/3157)	21.6 (681/3157)	25.7 (810/3157)	13.3 (421/3157)	7.9 (249/3157)		
	70–79	77.0 (1,318/1711)	46.37	<0.001	87.1 (1,491/1711)	5.73	0.057	29.9 (511/1711)	24.5 (420/1711)	22.7 (389/1711)	13.4 (229/1711)	9.5 (162/1711)	20.69	0.008
	80+	67.7 (417/616)			83.6 (515/616)			25.5 (157/616)	25.0 (154/616)	26.0 (160/616)	13.8 (85/616)	9.7 (60/616)		
Gender	Men	80.1 (2,153/2687)	17.41	<0.001	89.0 (2,392/2687)	24.84	<0.001	30.3 (814/2687)	22.1 (594/2687)	25.0 (673/2687)	13.0 (349/2687)	9.6 (257/2687)	8.07	0.089
	Women	75.4 (2,110/2797)			84.4 (2,362/2797)			30.4 (850/2797)	23.6 (661/2797)	24.5 (686/2797)	13.8 (386/2797)	7.7 (214/2797)		
Area ^d^	Urban	76.4 (1,639/2144)	3.38	0.066	86.7 (1858/2144)	0.002	0.961	29.9 (641/2144)	25.6 (548/2144)	22.2 (476/2144)	15.1 (324/2144)	7.2 (155/2144)	35.94	<0.001
	Suburban	78.6 (2,624/3340)			86.7 (2,896/3340)			30.6 (1,023/3340)	21.2 (707/3340)	26.4 (883/3340)	12.3 (411/3340)	9.5 (316/3340)		
Educational attainment (schooling years)	0–6	75.7 (2060/2721)			86.0 (2,340/2721)			29.4 (799/2721)	21.8 (593/2721)	25.6 (696/2721)	13.2 (358/2721)	10.1 (275/2721)		
	7-,12	79.5 (1960/2465)	13.46	0.001	87.2 (2,149/2465)	2.94	0.230	32.0 (788/2465)	24.1 (594/2465)	23.5 (579/2465)	13.6 (336/2465)	6.8 (168/2465)	28.55	<0.001
	13+	81.5 (243/298)			88.9 (256/298)			25.8 (77/298)	22.8 (68/298)	28.2 (84/298)	13.8 (41/298)	9.4 (28/298)		
Occupation ^e^	Domestic worker	82.2 (884/1075)			87.7 (943/1075)			29.7 (319/1075)	20.0 (215/1075)	29.8 (320/1075)	10.6 (114/1075)	10.0 (107/1075)		
	Industrial worker	74.0 (1722/2327)			86.2 (2006/2327)			30.2 (703/2327)	22.9 (532/2327)	20.8 (483/2327)	17.1 (399/2327)	9.0 (210/2327)		
	Service/sales worker	81.4 (275/338)	36.34	<0.001	86.7 (293/338)	6.03	0.303	32.8 (111/338)	17.2 (58/338)	29.6 (100/338)	14.8 (50/338)	5.6 (19/338)	129.63	<0.001
	Office worker	78.8 (349/443)			89.2 (395/443)			26.0 (115/443)	26.6 (118/443)	30.5 (135/443)	12.6 (56/443)	4.3 (19/443)		
	Academic/research staff	79.9 (307/384)			84.1 (323/384)			30.2 (116/384)	23.7 (91/384)	23.4 (90/384)	12.2 (47/384)	10.4 (40/384)		
	Self-employed	79.2 (726/917)			86.6 (794/917)			32.7 (300/917)	26.3 (241/917)	25.2 (231/917)	7.5 (69/917)	8.3 (76/917)		
Marital status	Single	68.7 (521/758)	41.18	<0.001	81.4 (617/758)	21.33	<0.001	27.3 (207/758)	23.0 (174/758)	23.2 (176/758)	17.8 (135/758)	8.7 (66/758)	16.30	0.003
	Married/having a partner	79.2 (3,742/4726)			87.5 (4,137/4726)			30.8 (1,457/4726)	22.9 (1,081/4726)	25.0 (1,183/4726)	12.7 (600/4726)	8.6 (405/4726)		
Smoking ^f^	No	77.4 (3,226/4168)	1.13	0.287	86.2 (3,591/4168)	4.26	0.039	30.3 (1,262/4168)	23.2 (966/4168)	25.1 (1,046/4168)	13.1 (548/4168)	8.3 (346/4168)	3.88	0.423
	Yes	78.8 (1,037/1316)			88.4 (1,163/1316)			30.5 (402/1316)	22.0 (289/1316)	23.8 (313/1316)	14.2 (187/1316)	9.5 (125/1316)		
Drinking ^g^	No	77.3 (3,689/4773)	4.24	0.040	86.3 (4,121/4773)	3.88	0.049	30.3 (1,444/4773)	23.1 (1,101/4773)	24.9 (1,189/4773)	13.5 (644/4773)	8.3 (395/4773)	5.30	0.258
	Yes	80.7 (574/711)			89.0 (633/711)			30.9 (220/711)	21.7 (154/711)	23.9 (170/711)	12.8 (91/711)	10.7 (76/711)		
Body weight status ^h^	BMI < 24	76.2 (1787/2346)			86.3 (2024/2346)			29.4 (690/2346)	22.5 (529/2346)	25.7 (604/2346)	13.6 (320/2346)	8.7 (203/2346)		
	24 ≤ BMI < 28	78.5 (1800/2293)	6.59	0.037	86.7 (1987/2293)	1.48	0.478	31.1 (713/2293)	22.5 (517/2293)	24.3 (558/2293)	13.3 (306/2293)	8.7 (199/2293)	4.95	0.763
	BMI ≥ 28	80.0 (676/845)			87.9 (743/845)			30.9 (261/845)	24.7 (209/845)	23.3 (197/845)	12.9 (109/845)	8.2 (69/845)		

a: PSQI-based sleep quality was categorized as “good (PSQI ≤7)” or “poor (PSQI >7).” b: Chi-square test. c: Self-rated sleep quality was classified as “good (excellent or good)” or “poor (poor or very poor).” d: Areas were categorized as urban and suburban districts based on the official classifications released by China National Bureau of Statistics. e: Occupation referred to job before retirement, and was classified for each participant based on recommendations by China National Center for Chronic Non-communicable Disease Prevention and Control. f: Smoking status was defined as smokers (current- and ex-smokers) and non-smokers (who never smoked cigarettes). g: Drinkers were defined as persons who drank alcohol, on average, at least two times a week for more than 1 year, while non-drinkers were those people who did not meet drinker’s definition. h: Body weight status was categorized based on cutoffs of body mass index (BMI) recommendations for Chinese adults.

Table 3 demonstrates the prevalence of fatigue among subjects in this study. For overall participants, the prevalence rates of physical and mental fatigue were 59.0% (95%CI = 57.7, 63.0) and 51.1% (95%CI = 49.8, 52.4), respectively. Interestingly, for the distribution of fatigue by the six aforementioned socio-demographic characteristics, the prevalence of either physical or mental fatigue was different by age, education, and marriage. Particularly, among those aged 60–69, 70–79 and 80 + years, the prevalence was, respectively, 56.5% (95%CI = 54.8, 58.2), 60.1% (95%CI = 57.7, 62.4) and 68.0% (95%CI = 64.1, 71.6) for physical fatigue, and 49.1% (95%CI = 47.3, 50.9), 51.9% (95%CI = 49.5, 54.3) and 59.4% (95%CI = 55.4, 63.3), for mental fatigue, separately.

**Table 3:** The prevalence of fatigue among men and women aged 60 + years by selected characteristics in this study (*N* = 5,484).

Characteristics		% (n/N) of participants with fatigability					
		Higher physical fatigability (PFS ≥ 15)^a^	*χ* ^2^	*p*-value^b^	Higher mental fatigability (PFS ≥ 13)^a^	*χ* ^2^	*p-*value^b^
Overall		59.0 (3,233/5484)			51.1 (2,803/5484)		
Age (years)	60–69	56.5 (1785/3157)			49.1 (1,549/3157)		
	70–79	60.1 (1,029/1711)	29.51	<0.001	51.9 (888/1711)	22.71	<0.001
	80+	68.0 (419/616)			59.4 (366/616)		
Gender	Men	57.9 (1,556/2687)	2.38	0.123	50.5 (1,356/2687)	0.88	0.347
	Women	60.0 (1,677/2797)			51.7 (1,447/2797)		
Area ^c^	Urban	58.8 (1,260/2144)	0.05	0.824	52.5 (1,125/2144)	2.61	0.107
	Suburban	59.1 (1973/3340)			50.2 (1,678/3340)		
Educational attainment (schooling years)	0–6	61.6 (1,675/2721)			52.7 (1,435/2721)		
	7-,12	55.6 (1,371/2465)	20.73	<0.001	48.8 (1,202/2465)	10.84	0.004
	13+	62.8 (187/298)			55.7 (166/298)		
Occupation ^d^	Domestic worker	62.3 (670/1075)			54.1 (582/1075)		
	Industrial worker	58.3 (1,357/2327)			51.0 (1,187/2327)		
	Service/sales worker	60.4 (204/338)	5.78	0.181	49.1 (166/338)	6.43	0.266
	Office worker	58.2 (258/443)			51.5 (228/443)		
	Academic/research staff	57.8 (222/384)			49.2 (189/384)		
	Self-employed	56.9 (522/917)			49.2 (451/917)		
Marital status	Single	63.6 (482/758)	7.81	0.005	55.3 (419/758)	6.11	0.013
	Married/having a partner	58.2 (2,751/4726)			50.4 (2,384/4726)		
Smoking ^e^	No	59.2 (2,468/4168)	0.48	0.487	51.3 (2,140/4168)	0.37	0.542
	Yes	58.1 (765/1316)			50.4 (663/1316)		
Drinking ^f^	No	59.6 (2,847/4773)	7.34	0.007	51.9 (2,479/4773)	10.04	0.002
	Yes	54.3 (386/711)			45.6 (324/711)		
Body weight status ^g^	BMI < 24	58.6 (1,374/2346)			51.1 (1,199/2346)		
	24 ≤ BMI < 28	58.9 (1,350/2293)	0.73	0.696	51.1 (1,172/2293)	0.00	1.000
	BMI ≥ 28	60.2 (509/845)			51.1 (432/845)		

a: Fatigability was assessed using Pittsburgh Fatigability Scale (PFS), with PFS≥15 and 1 ≥ 3 indicating high physical and mental fatigability, respectively. b: Chi-square test. c: Areas were categorized as urban or suburban districts based on the official classifications released by China National Bureau of Statistics. d: Occupation referred to job before retirement, and was classified for each participant based on recommendations by China National Center for Chronic Non-communicable Disease Prevention and Control. e: Smoking status was defined as smokers (current- and ex-smokers) and non-smokers (who never smoked cigarettes). f: Drinkers were defined as persons who drank alcohol, on average, at least two times a week for more than 1 year, while non-drinkers were those people who did not meet drinker’s definition. g: Body weight status was categorized based on cutoffs of body mass index (BMI) recommendations for Chinese adults.

Table 4 presents the associations of PSQI-based sleep quality with physical and mental fatigue among participants. Among good and poor PSQI-based sleepers, the prevalence was 55.6% (95%CI = 54.1, 57.1) and 70.7% (95%CI = 68.0, 73.2) separately for physical fatigue, and 47.8% (95%CI = 46.3, 49.3) and 62.6% (95%CI = 59.8, 65.3) respectively for mental fatigue. For overall participants, after control for potential confounding factors, those with good PSQI-based sleep quality were less likely to report physical (OR = 0.61, 95%CI = 0.53, 0.70) and mental fatigue (OR = 0.65, 95%CI = 0.56, 0.74) compared to their counterparts with poor PSQI-based sleep quality. Meaningfully, the negative association of PSQI-based sleep quality with either physical or mental fatigue was also observed among each stratum of participants by age, gender or residence area.

**Table 4:** The relationship between PSQI-based sleep quality and fatigue among men and women aged 60 + years in this study (*N* = 5,484).

Characteristics		PSQI-based sleep quality^b^	OR (95%CI) for experiencing fatigue^a^					
			% (n/N) of physical fatigue^c^	Model 1^†^	Model 2^‡^	% (n/N) of mental fatigue^c^	Model 1^†^	Model 2^‡^
Overall		Poor	70.7 (863/1221)	1	1	62.6 (764/1221)	1	1
		Good	55.6 (2,370/4263)	0.52 (0.45, 0.60)	0.61 (0.53, 0.70)	47.8 (2039/4263)	0.55 (0.48, 0.63)	0.65 (0.56, 0.74)
Age (years)	60–69	Poor	66.9 (421/629)	1	1	58.2 (366/629)	1	1
		Good	54.0 (1,364/2528)	0.58 (0.48, 0.70)	0.63 (0.52, 0.77)	46.8 (1,183/2528)	0.63 (0.53, 0.75)	0.72 (0.59, 0.86)
	70–79	Poor	71.0 (279/393)	1	1	64.6 (254/393)	1	1
		Good	56.9 (750/1318)	0.54 (0.42, 0.69)	0.61 (0.47, 0.79)	48.1 (634/1318)	0.51 (0.40, 0.64)	0.57 (0.45, 0.73)
	80+	Poor	81.9 (163/199)	1	1	72.4 (144/199)	1	1
		Good	61.4 (256/417)	0.35 (0.23, 0.53)	0.46 (0.29, 0.72)	53.2 (222/417)	0.44 (0.30, 0.63)	0.58 (0.39, 0.86)
Gender	Men	Poor	70.4 (376/534)	1	1	62.9 (336/534)	1	1
		Good	54.8 (1,180/2153)	0.51 (0.42, 0.63)	0.61 (0.50, 0.76)	47.4 (1,020/2153)	0.53 (0.44, 0.65)	0.66 (0.53, 0.81)
	Women	Poor	70.9 (487/687)	1	1	62.3 (428/687)	1	1
		Good	56.4 (1,190/2110)	0.53 (0.44, 0.64)	0.60 (0.49, 0.73)	48.3 (1,019/2110)	0.57 (0.47, 0.67)	0.64 (0.53, 0.78)
Area	Urban	Poor	68.9 (348/505)	1	1	62.6 (316/505)	1	1
		Good	55.6 (912/1639)	0.57 (0.46, 0.70)	0.66 (0.52, 0.82)	49.4 (809/1639)	0.58 (0.48, 0.72)	0.70 (0.56, 0.87)
	Sub-urban	Poor	71.9 (515/716)	1	1	62.6 (448/716)	1	1
		Good	55.6 (1,458/2624)	0.49 (0.41, 0.58)	0.57 (0.47, 0.69)	46.9 (1,230/2624)	0.53 (0.45, 0.63)	0.62 (0.52, 0.74)

a: OR = odds ratio, CI = confidence interval. b: PSQI-based sleep quality was categorized as “good (PSQI ≤7)” or “poor (PSQI >7).” c: Fatigue was assessed using Pittsburgh Fatigability Scale (PFS), with PFS≥15 and 13 indicating high physical and mental fatigability, respectively. † Model 1 was an unadjusted mixed-effect logistic regression model with PSQI sleep quality as the single predictor and adjustment for neighborhood-level clustering effects. ‡ Model 2 was a multivariate mixed-effect logistics regression model with adjustment for age (where applicable), gender (where applicable), educational attainment, marital status, occupation, body weight status, smoking, drinking, physical activity, meat consumption, vegetable consumption, self-reported histories of main chronic diseases (diabetes, hypertension, COPD, stroke, coronary heart disease, kidney disease, cancer, abnormal lipid profile), frailty, depression, and neighborhood-level clustering effects in addition to those included in Model 1.

Table 5 illustrates the relationship between self-rated sleep quality and fatigue among participants. Within good and poor self-rated sleepers, the prevalence was 58.0% (95%CI = 56.6 59.4) and 65.1% (95%CI = 61.5, 68.5) separately for physical fatigue, and 50.3% (95%CI = 48.9, 51.7) and 56.4% (95%CI = 52.7, 60.0) respectively for mental fatigue. Different from the scenario of significant associations between PSQI-based sleep quality and fatigue, there was no significant link between self-rated sleep quality and either physical (OR = 0.88, 95%CI = 0.74, 1.04) or mental fatigue (OR = 0.92, 95%CI = 0.78, 1.09) among overall participants after adjustment for confounders. Moreover, self-rated sleep quality was also not in relation to both physical and mental fatigue among subjects by age, gender, or area, with an exception of that significantly negative association existed between self-rated sleep quality and either physical (OR = 0.59; 95%CI = 0.43, 0.82) or mental fatigue (OR = 0.62; 95%CI = 0.46, 0.85) among participants aged 70–79 years.

**Table 5:** The relationship between self-rated sleep quality and fatigue among men and women aged 60+ years in this study (*N* = 5,484).

Characteristics		Self-rated sleep quality ^b^	OR (95%CI) for experiencing fatigue^a^					
			% (n/N) of physical fatigue^c^	Model 1^†^	Model 2^‡^	% (n/N) of mental fatigue^c^	Model 1^†^	Model 2^‡^
Overall		Poor	65.1 (475/730)	1	1	56.4 (412/730)	1	1
		Good	58.0 (2,758/4754)	0.74 (0.63, 0.87)	0.88 (0.74, 1.04)	50.3 (2,391/4754)	0.78 (0.67, 0.91)	0.92 (0.78, 1.09)
Age (years)	60–69	Poor	57.9 (237/409)	1	1	49.6 (203/409)	1	1
		Good	56.3 (1,548/2748)	0.94 (0.76, 1.16)	1.08 (0.86, 1.35)	49.0 (1,346/2748)	0.97 (0.79, 1.20)	1.12 (0.90, 1.40)
	70–79	Poor	73.2 (161/220)	1	1	65.0 (143/220)	1	1
		Good	58.2 (868/1491)	0.51 (0.37, 0.70)	0.59 (0.43, 0.82)	50.0 (745/1491)	0.54 (0.40, 0.72)	0.62 (0.46, 0.85)
	80+	Poor	76.2 (77/101)	1	1	65.3 (66/101)	1	1
		Good	66.4 (342/515)	0.62 (0.38, 1.01)	0.73 (0.44, 1.23)	58.3 (300/515)	0.74 (0.47, 1.16)	0.89 (0.55, 1.42)
Gender	Men	Poor	65.4 (193/295)	1	1	57.6 (170/295)	1	1
		Good	57.0 (1,363/2392)	0.70 (0.54, 0.90)	0.86 (0.66, 1.12)	49.6 (1,186/2392)	0.72 (0.57, 0.93)	0.89 (0.69, 1.15)
	Women	Poor	64.8 (282/435)	1	1	55.6 (242/435)	1	1
		Good	59.1 (1,395/2362)	0.78 (0.63, 0.97)	0.89 (0.71, 1.11)	51.0 (1,205/2362)	0.83 (0.68, 1.02)	0.95 (0.76, 1.18)
Area	Urban	Poor	66.4 (190/286)	1	1	59.4 (170/286)	1	1
		Good	57.6 (1,070/1858)	0.69 (0.53, 0.89)	0.79 (0.56, 1.04)	51.4 (955/1858)	0.72 (0.56, 0.93)	0.84 (0.64, 1.10)
	Sub-urban	Poor	64.2 (285/444)	1	1	54.5 (242/444)	1	1
		Good	58.3 (1,688/2896)	0.78 (0.63, 0.96)	0.94 (0.76, 1.17)	49.6 (1,436/2896)	0.82 (0.67, 1.002)	0.98 (0.79, 1.21)

a: OR = odds ratio, CI = confidence interval. b: Perceived sleep quality was classified as “good (excellent or good)” or “poor (poor or very poor).” c: Fatigue was assessed using Pittsburgh Fatigability Scale (PFS), with PFS≥15 and ≥13 indicating high physical and mental fatigability, respectively. † Model 1 was an unadjusted mixed-effect logistic regression model with perceived sleep quality as the single predictor and adjustment for neighborhood-level clustering effects. ‡ Model 2 was a multivariate mixed-effect logistics regression model with adjustment for age (where applicable), gender (where applicable), educational attainment, marital status, occupation, body weight status, smoking, drinking, physical activity, meat consumption, vegetable consumption, self-reported histories of main chronic diseases (diabetes, hypertension, COPD, stroke, coronary heart disease, kidney disease, cancer, abnormal lipid profile), frailty, depression, and neighborhood-level clustering effects in addition to those included in Model 1.

Table 6 elucidates the association between daily sleep duration and fatigue in study subjects. Among participants with a sleep duration of <6.0, 6.0–6.9, 7.0–7.9, 8.0–8.9 and ≥9.0 h/day, the respective prevalence was 65.7% (95%CI = 62.1, 69.1), 63.3% (95%CI = 60.6, 66.0), 53.4% (95%CI = 51.0, 55.8), 56.3% (95%CI = 53.6, 59.0) and 63.9% (95%CI = 59.4, 68.2) for physical fatigue, while 59.7% (95%CI = 56.0, 63.3), 55.4% (95%CI = 52.6, 58.2), 45.7% (95%CI = 43.3, 48.1), 47.8% (95%CI = 45.1, 50.5) and 54.8% (95%CI = 50.2, 59.3) for mental fatigue. With consideration of potential confounders, a U-shape association of sleep duration with either physical or mental fatigue was examined among overall participants. The odds were 1.52 (95%CI = 1.26, 1.83), 1.47 (95%CI = 1.26, 1.72), 1.09 (95%CI = 0.94, 1.26), and 1.46 (95%CI = 1.17, 1.81) for experiencing physical fatigue, and 1.60 (95%CI = 1.33, 1.92), 1.43 (95%CI = 1.23, 1.66), 1.07 (95%CI = 0.92, 1.23), and 1.37 (95%CI = 1.11, 1.69) for reporting mental fatigue, respectively, among participants with a sleep duration of <6.0, 6.0–6.9, 8.0–8.9, and ≥9.0 h/day relative to those with the sleep duration of 7.0–7.9 h/day. Moreover, the scenarios of relationship between sleep duration and fatigue among subjects aged 60–69 years, men, or sub-urban residents were the same as that observed in overall participants.

**Table 6:** The relationship between sleep time and fatigue among men and women aged 60 + years in this study (*N* = 5,484).

Characteristics		Sleep time^b^	OR (95%CI) for experiencing fatigue^a^					
			% (n/N) of physical fatigue^c^	Model 1^†^	Model 2^‡^	% (n/N) of mental fatigue^c^	Model 1^†^	Model 2^‡^
Overall		<6.0 h/d	65.7 (483/735)	1.67 (1.40, 2.00)	1.52 (1.26, 1.83)	59.7 (439/735)	1.76 (1.48, 2.10)	1.60 (1.33, 1.92)
		6.0–6.9 h/d	63.3 (795/1255)	1.51 (1.30, 1.75)	1.47 (1.26, 1.72)	55.4 (695/1255)	1.47 (1.27, 1.71)	1.43 (1.23, 1.66)
		7.0–7.9 h/d	53.4 (889/1664)	1	1	45.7 (761/1664)	1	1
		8.0–8.9 h/d	56.3 (765/1359)	1.12 (0.97, 1.30)	1.09 (0.94, 1.26)	47.8 (650/1359)	1.09 (0.94, 1.26)	1.07 (0.92, 1.23)
		≥9.0 h/d	63.9 (301/471)	1.54 (1.25, 1.91)	1.46 (1.17, 1.81)	54.8 (258/471)	1.44 (1.17, 1.77)	1.37 (1.11, 1.69)
Age (years)	60–69	<6.0 h/d	61.5 (259/421)	1.43 (1.14, 1.81)	1.38 (1.09, 1.76)	55.3 (233/421)	1.49 (1.18, 1.87)	1.40 (1.10, 1.77)
		6.0–6.9 h/d	59.9 (408/681)	1.34 (1.10, 1.63)	1.36 (1.11, 1.66)	51.8 (353/681)	1.29 (1.06, 1.57)	1.28 (1.05, 1.56)
		7.0–7.9 h/d	52.7 (525/996)	1	1	45.5 (453/996)	1	1
		8.0–8.9 h/d	53.8 (436/810)	1.05 (0.87, 1.26)	1.04 (0.86, 1.26)	46.4 (376/810)	1.04 (0.86, 1.25)	1.04 (0.86, 1.25)
		≥9.0 h/d	63.1 (157/249)	1.53 (1.15, 2.04)	1.52 (1.14, 2.04)	53.8 (134/249)	1.40 (1.06, 1.85)	1.37 (1.03, 1.82)
	70–79	<6.0 h/d	69.4 (159/229)	2.06 (1.48, 2.87)	1.76 (1.25, 2.49)	66.4 (152/229)	2.51 (1.81, 3.47)	2.20 (1.57, 3.08)
		6.0–6.9 h/d	66.9 (281/420)	1.83 (1.40, 2.40)	1.76 (1.34, 2.32)	58.8 (247/420)	1.82 (1.40, 2.36)	1.75 (1.34, 2.29)
		7.0–7.9 h/d	52.4 (268/511)	1	1	44.0 (225/511)	1	1
		8.0–8.9 h/d	56.6 (220/389)	1.18 (0.91, 1.54)	1.07 (0.81, 1.41)	46.5 (181/389)	1.11 (0.85, 1.44)	1.01 (0.76, 1.32)
		≥9.0 h/d	62.3 (101/162)	1.50 (1.05, 2.16)	1.31 (0.90, 1.92)	51.2 (83/162)	1.34 (0.94, 1.90)	1.19 (0.82, 1.71)
	80+	<6.0 h/d	76.5 (65/85)	2.07 (1.14, 3.75)	1.80 (0.95, 3.43)	63.5 (54/85)	1.55 (0.90, 2.67)	1.34 (0.74, 2.41)
		6.0–6.9 h/d	68.8 (106/154)	1.40 (0.88, 2.24)	1.57 (0.94, 2.62)	61.7 (95/154)	1.44 (0.91, 2.26)	1.58 (0.97, 2.60)
		7.0–7.9 h/d	61.1 (96/157)	1	1	52.9 (83/157)	1	1
		8.0–8.9 h/d	68.1 (109/160)	1.36 (0.86, 2.16)	1.45 (0.88, 2.39)	58.1 (93/160)	1.24 (0.79, 1.93)	1.33 (0.82, 2.15)
		≥9.0 h/d	71.7 (43/60)	1.61 (0.84, 3.07)	1.79 (0.89, 3.60)	68.3 (41/60)	1.92 (1.03, 3.60)	2.18 (1.11, 4.29)
Gender	Men	<6.0 h/d	62.8 (219/349)	1.60 (1.24, 2.07)	1.48 (1.13, 1.92)	56.2 (196/349)	1.65 (1.28, 2.12)	1.49 (1.15, 1.94)
		6.0–6.9 h/d	64.6 (384/594)	1.74 (1.40, 2.16)	1.71 (1.37, 2.14)	56.7 (337/594)	1.69 (1.36, 2.09)	1.62 (1.30, 2.02)
		7.0–7.9 h/d	51.2 (417/814)	1	1	43.7 (356/814)	1	1
		8.0–8.9 h/d	55.3 (372/673)	1.18 (0.96, 1.44)	1.19 (0.97, 1.47)	47.8 (322/673)	1.18 (0.96, 1.45)	1.19 (0.97, 1.47)
		≥9.0 h/d	63.8 (164/257)	1.68 (1.26, 2.24)	1.66 (1.24, 2.23)	56.4 (145/257)	1.67 (1.26, 2.21)	1.66 (1.24, 2.21)
	Women	<6.0 h/d	68.4 (264/386)	1.73 (1.35, 2.23)	1.57 (1.21, 2.05)	63.0 (243/386)	1.87 (1.46, 2.39)	1.71 (1.32, 2.21)
		6.0–6.9 h/d	62.2 (411/661)	1.32 (1.07, 1.62)	1.28 (1.03, 1.58)	54.2 (358/661)	1.30 (1.06, 1.59)	1.25 (1.01, 1.54)
		7.0–7.9 h/d	55.5 (472/850)	1	1	47.6 (405/850)	1	1
		8.0–8.9 h/d	57.3 (393/686)	1.07 (0.88, 1.32)	1.01 (0.81, 1.24)	47.8 (328/686)	1.01 (0.82, 1.23)	0.95 (0.77, 1.17)
		≥9.0 h/d	64.0 (137/214)	1.43 (1.05, 1.94)	1.27 (0.92, 1.75)	52.8 (113/214)	1.23 (0.91, 1.66)	1.13 (0.82, 1.52)
Area	Urban	<6.0 h/d	60.2 (195/324)	1.20 (0.92, 1.58)	1.05 (0.78, 1.40)	56.2 (182/324)	1.29 (0.99, 1.69)	1.09 (0.82, 1.45)
		6.0–6.9 h/d	62.8 (344/548)	1.34 (1.06, 1.69)	1.32 (1.04, 1.68)	57.3 (314/548)	1.35 (1.08, 1.70)	1.33 (1.05, 1.68)
		7.0–7.9 h/d	55.7 (357/641)	1	1	49.8 (319/614)	1	1
		8.0–8.9 h/d	56.7 (270/476)	1.04 (0.82, 1.32)	1.01 (0.79, 1.29)	47.9 (228/476)	0.93 (0.73, 1.18)	0.90 (0.71, 1.16)
		≥9.0 h/d	60.6 (94/155)	1.23 (0.86, 1.75)	1.13 (0.78, 1.64)	52.9 (82/155)	1.13 (0.80, 1.61)	1.06 (0.74, 1.53)
	Sub-urban	<6.0 h/d	70.1 (288/411)	2.16 (1.69, 2.76)	1.95 (1.51, 2.51)	62.5 (257/411)	2.19 (1.73, 2.78)	1.99 (1.56, 2.54)
		6.0–6.9 h/d	63.8 (451/707)	1.63 (1.34, 1.98)	1.59 (1.30, 1.95)	53.9 (381/707)	1.54 (1.27, 1.86)	1.50 (1.23, 1.82)
		7.0–7.9 h/d	52.0 (532/1023)	1	1	43.2 (442/1023)	1	1
		8.0–8.9 h/d	56.1 (495/883)	1.18 (0.98, 1.41)	1.14 (0.95, 1.37)	47.8 (422/883)	1.20 (1.01, 1.44)	1.17 (0.97, 1.40)
		≥9.0 h/d	65.5 (207/316)	1.75 (1.35, 2.28)	1.56 (1.22, 2.09)	55.7 (176/316)	1.65 (1.28, 2.13)	1.52 (1.17, 1.98)

a: OR = odds ratio, CI = confidence interval. b: Perceived sleep quality was classified as “good (excellent or good)” or “poor (poor or very poor).” c: Fatigue was assessed using Pittsburgh Fatigability Scale (PFS), with PFS≥15 and ≥13 indicating high physical and mental fatigability, respectively. † Model 1 was an unadjusted mixed-effect logistic regression model with perceived sleep quality as the single predictor and adjustment for neighborhood-level clustering effects. ‡ Model 2 was a multivariate mixed-effect logistics regression model with adjustment for age (where applicable), gender (where applicable), educational attainment, marital status, occupation, body weight status, smoking, drinking, physical activity, meat consumption, vegetable consumption, self-reported histories of main chronic diseases (diabetes, hypertension, COPD, stroke, coronary heart disease, kidney disease, cancer, abnormal lipid profile), frailty, depression, and neighborhood-level clustering effects in addition to those included in Model 1.

## Discussion

In this population study, the main aims were to examine the associations of sleep quality and duration with physical and mental fatigue, separately, among representative community-residing older participants in Nanjing municipality of China. It was investigated that, among older adults aged 60 years or above in regional China, PSQI-based, not self-rated, sleep quality was negatively associated with both physical and mental fatigue, and a U-shape relationship was observed between daily sleep duration and physical/mental fatigue.

Strictly speaking, it is really difficult to make comparison of the findings in previous literature and this study, as they were different in terms of study design [cross-sectional survey (10, 17), follow-up investigation (15, 16), or cohort study (14)], participants [individuals with chronic conditions (14), special subjects (medicare beneficiaries) (15), people with good health (16), or community-dwellers (10, 17)], sleep assessment [PSQI (10, 14), specific questions (15, 17), objective device (16)], or fatigue measurement [PFS (14, 16), Persistent severe fatigue (15), the Piper Fatigue Scale (17), the Patient-Reported Outcomes Measurement Information System (10)]. On the other hand, the present study was designed as a cross-sectional survey with participants representatively selected from overall older residents aged 60 + years in Nanjing municipality of China. Moreover, fatigue was measured using PFS, while sleep attributes were assessed separately with both PSQI-based/self-rated sleep quality and typically daily sleep duration. However, in a broad sense, the main findings in this study were consistent with those reported in the existing investigations that sleep quality was negatively associated with fatigue and a U-shape relationship was found between sleep duration and fatigue (10, 14–17).

Interestingly, only sleep quality assessed with PSQI was associated with fatigue, while no link was examined between self-rated sleep quality and fatigue in this study. This may be largely explained by the difference in question items involved in PSQI-based and self-rated approaches to assess sleep quality. Although both PSQI-based and self-rated approaches assess sleep quality relying on participant’s subjective response to question items, PSQI comprises seven domains and 19 specific qualitative and particularly quantitative items (28–30). Moreover, an integrated index is created to reflect sleep quality based on the total score calculated from those assigned to each of the 19 items, which means that the sleep quality measured with PSQI is not only comprehensive but also relatively-stable for participants. On the other hand, the self-rated sleep quality was assessed simply using one subjective question item (“How did you feel about your sleep quality under the usual circumstance in the past month?. The 4-level Likert answer option is: excellent, good, poor, or very poor” (28–30)). Clearly, this is a purely qualitative measurement of sleep quality based on subjective response to one single psychometric question, which implies that the sleep quality assessed in this way may be occasional and unstable. Potentially, these may be the reasons behind disparities in associations of PSQI-assessed and self-rated sleep quality with fatigue among participants in this study. Notably, this finding is meaningful for clinical and public health practice, suggesting that one-item-based self-rated sleep quality may not be suitable for identifying fatigue among older adults in China.

The following mechanisms may underlie the negative relationship between sleep quality and fatigue. Firstly, good sleep quality usually means less disturbance of sleep, while, contrarily, poor sleep quality implies more sleep disturbance, and disturbance of sleep is one of the major risk factors for fatigue (14, 17). Secondly, it may also be explained biologically by that poor sleep quality can induce abnormal hypothalamus-pituitary–adrenal axis and pro-inflammatory cytokine levels for people, and these two abnormal conditions are associated with fatigue (44–46). With respect to the U-shape relationship between sleep duration and fatigue, it is not easy to explain clearly. In the previous study identified a U-shape relationship between sleep duration and fatigue, the mechanism was not discussed (17). Actually, different from poor sleep quality, either short or long sleep duration does not definitely imply disturbance of sleep. For the negative relationship between short sleep duration and fatigue, it is relatively understandable that regular deprivation of sleep time may cause tiredness or/and burnout, and subsequently leads to fatigue (47, 48). The real challenge is how to explain the positive association of long sleep duration with fatigue. One possible assumption is that the longer time does not warrant a refreshing or restorative sleep for older adults, and thus the long-duration sleeper may not be recuperative and energetic after sleep.

This is the first study to investigate the associations of PSQI-based and self-rated sleep quality, sleep duration with both physical and mental fatigue among community-dwelling older residents in regional China. Some strengths are worthy of mention. Firstly, participants were randomly selected from community-residing older inhabitants within Nanjing municipality of China. Moreover, these study subjects were well representative of the total 1.95 million local men and women aged 60 years or above in Nanjing (19). Secondly, three measures of sleep attributes (PSQI-based and self-rated sleep quality, and sleep duration) were employed to investigate association of sleep with physical and mental fatigue in an individual study. Thirdly, fatigue was measured with the validated Chinese version of PFS, a well-accepted instrument for assessing fatigue with consideration of specific standardized daily activities. Fourthly, analysis was conducted with full adjustment for potential confounders, including participants’ socio-demographic attributes and lifestyle/behaviors, fatigue-inducing chronic conditions, frailty, depressive symptoms, and community-level clustering effects. The last, findings were interesting and meaningful. PSQI-based, not self-rated, sleep quality was in negative relation to both physical and mental fatigue, and a U-shape relationship was examined between sleep duration and fatigue among older community-residing Chinese adults.

However, there were several limitations in the present study. First, no causal relationship between sleep quality/duration and fatigue could be concluded from the present study due to the nature of cross-sectional study design. Next, data on fatigue and sleep were self-assessed using questionnaires by participants, although these questionnaires/instruments were validated. Third, as PSQI has been examined with a cutoff value of >7 performing better for screening older adults with sleep problem in China (28, 29), this Chinese-specific cutoff was employed to identify older adults with sleep problem in this study. However, it implies that the prevalence of sleep problem may be under-estimated and not comparable to those determined in studies using the original PSQI cutoff of >5 points. Fourth, participants’ lifestyle and behavior, fatigue-inducing chronic conditions were also self-reported, which may also imply potential bias of recall. Hence, the findings in the study shall be interpreted prudently. In future, longitudinal/intervention studies are encouraged to further examine the associations of sleep quality and duration with fatigue among community-residing older residents.

In conclusion, PSQI-based, not self-rated, sleep quality was negatively associated with fatigue, and a U-shape relationship was examined between sleep duration and fatigue among older community-dwelling people in regional China. This study has significant public health implications in this rapidly-aging world for building a healthy-aging society, suggesting that population-level fatigue may be improved through interventions of healthy sleep.

## Data Availability

The original contributions presented in the study are included in the article/supplementary material, further inquiries can be directed to the corresponding author/s.
